# Effect of the vascular endothelial growth factor expression level on angiopoietin-2-mediated nasopharyngeal carcinoma growth

**DOI:** 10.1186/2045-824X-6-4

**Published:** 2014-03-01

**Authors:** Hai-Hong Chen, Bin-Qi Weng, Ke-Jia Cheng, Hong-Yan Liu, Shen-Qing Wang, Yv-Yv Lu

**Affiliations:** 1 Department of Head-neck Otolaryngology, The First Affiliated Hospital, College of Medicine, Zhejiang University, QingChun Road 79, Hangzhou 310003, China

**Keywords:** Angiopoietin-2, Vascular endothelial growth factor, Nasopharyngeal carcinoma, Tumor angiogenesis

## Abstract

**Background:**

The overexpression of angiopoietin-2 (Ang-2) has both pro-tumorigenic and anti-tumorigenic effects. However, the mechanisms of this protein’s dual effects are poorly understood, and it remains unclear how Ang-2 cooperates with vascular endothelial growth factor (VEGF). In the current study, we investigated the effects of Ang-2 overexpression on nasopharyngeal carcinoma growth in the presence of different levels of VEGF.

**Methods:**

Ang-2 was introduced into the CNE2 cell line by liposome transfection, and the expression of endogenous VEGF was inhibited by microRNA-mediated RNA interference. CNE2 cells expressing varying levels of Ang-2 and VEGF were injected subcutaneously into the flanks of nude mice. Tumor growth was measured, and vessels from the harvested tumors were analyzed.

**Results:**

The overexpression of Ang-2 had no obvious effect on CNE2 tumor growth in the presence of endogenous VEGF but significantly inhibited CNE2 tumor growth when the expression of endogenous VEGF was silenced, and the Ang-2/VEGF ratio is negatively correlated with tumor growth. Ang-2 overexpression decreased the percentage of α-SMA-positive cells around the tumor vessels but reduced the microvessel density only in the absence of VEGF.

**Conclusions:**

Our results indicate that the effects of Ang-2 on nasopharyngeal carcinoma are highly dependent on the level of VEGF expression, Ang-2/VEGF ratio may offer a novel therapeutic approach for treating human cancer.

## Background

The development and growth of solid tumors depend on the pathological formation of new blood vessels. Over the last decade, much research has focused on elucidating the important role of angiopoietin-2 (Ang-2) in tumor angiogenesis. Ang-2, a member of the Ang family, acts as an agonist in Tie2 signaling and disrupts the integrity of mature vessels. The overexpression of Ang-2 has both pro-tumorigenic [1-6] and anti-tumorigenic effects [7-10]. The molecular mechanisms of the dual properties of Ang-2 are poorly understood. The effects of Ang-2 are highly context dependent and are associated with the level of vascular endothelial growth factor (VEGF). One possible explanation is that Ang-2 destabilizes blood vessels by disrupting the interactions between endothelial cells (ECs) and peri-ECs, causing Ang-2 to accelerate vessel regression in the absence of VEGF. However, in the presence of VEGF, Ang-2 enhances VEGF stimulation and promotes the sprouting of new vessels from disrupted vessels. Indeed, in non-small cell lung cancer patients, an angiogenic effect of Ang-2 was seen only when VEGF expression was high [11]. According to this hypothesis, the overexpression of Ang-2 and the inhibition of VEGF may have a complementary effect on the suppression of tumor growth. However, some studies have shown that Ang-2 limited the antivascular effects of VEGF inhibition [12]. Research performed by Hashizume [13] yielded the same result, indicating that inhibiting the expression of both Ang-2 and VEGF had additive effects on sprouting and vessel regression, resulting in the further slowing of tumor growth. The complexities of the function of Ang-2 are highly cell context dependent and have not been adequately explored. It remains unclear how Ang-2 and VEGF cooperate. An improved understanding of the roles of Ang-2 in tumor angiogenesis and the cooperation of Ang-2 with VEGF will lead to advances in the development of effective anti-angiogenic and anti-cancer therapies.

In this study, to gain insight into the dual roles of Ang-2 and its potential connection to VEGF, towards the ultimate goal of enhancing the efficacy of Ang-2-based therapeutic interventions, we investigated how Ang-2 overexpression influences tumor growth in the presence of different levels of VEGF. Nasopharyngeal carcinoma (NPC) is one of the most common cancers in Southern China, South Asia, and North Africa [14,15]. It is highly invasive and metastatic. The standard treatment for NPC is radiotherapy; however, the survival rate remains low. Angiogenesis is closely correlated with the progression of NPC and the effect of radiation therapy [16,17]. Accumulating evidence suggests that the overexpression of VEGF is positively correlated with tumor growth, metastatic potential, the failure of radiotherapy, and a poor prognosis [18,19]. However, limited evidence suggests that Ang-2 plays a role in NPC. We evaluated the combined effects of Ang-2 and VEGF on the angiogenic activity of nasopharyngeal carcinoma, and concluded that Ang-2 can suppress tumor growth and angiogenesis when the VEGF level is low, and the Ang-2/VEGF ratio is negatively correlated with tumor growth.

## Materials and methods

### Cell lines and culture conditions

The CNE2 human nasopharyngeal carcinoma cell line was obtained from the cell bank of the Shanghai Institute of Biochemistry and Cell Biology. The cells were cultured and maintained in RPMI 1640 medium (GIBCO) supplemented with 10% fetal bovine serum, 100 IU/mL penicillin, 100 mg/mL streptomycin, and 2 mM L-glutamine at 37°C in 5% CO_2_ and 95% air.

### Transfection of CNE2 cells with VEGF-specific miRNAs

Pre-miRNA oligo sequences for VEGFA (Gene ID:7422) were designed by Invitrogen’s Oligo Designer3.0 to target the gene sequence 5’-AACCATGAACTTTCTGCTGT-3’ (bases 1034–1054). The synthesized complementary DNA oligos targeting VEGF (forward oligo: 5’-TGCTGGACAGCAGAAAGTTCATGGTTGTTTTGGCCACTGACTGACAACCATGATTTCTGCTGTC-3’, reverse oligo: 5’-CCTGGACAGCAGAAATCATGGTTGTCAGTCAGTGGCCAAAACAACCATGAACTTTCTGCTGTCC-3’) or targeting a random sequence (forward oligo: 5’-TGCTGAAATGTACTGCGCGTGGAGACGTTTTGGCCACTGACTGACGTCTCCACGCAGTACATTT-3’, reverse oligo: 5’-CCTGAAATGTACTGCGTGGAGACGTCAGTCAGTGGCCAAAACGTCTCCACGCGCAGTACATTTC-3’) were annealed to generate a double-stranded oligo and cloned into the linearized pcDNA™ 6.2-GW/EmGFP-miR vector (Invitrogen, catalog no. K4936-00) using T4 DNA ligase. All of the vectors were transformed into DH5α Chemically Competent E. coli (Invitrogen Corp.), and the colonies containing spectinomycin-resistant transformants were analyzed for the desired expression clones. The recombinant vectors were purified with an Endotoxin-free plasmid DNA purification kit (NucleoBond® Xtra Midi EF) and confirmed by sequencing. The purified vectors were transfected into CNE2 using Lipofectamine 2000 (Invitrogen, catalog no. 11668-027) according to the manufacturer’s instructions, and cell lines stably integrating the pre-miRNA oligo sequences were selected out by Blasticidin S HCl (Invitrogen, catalog no. R210-01). Once transcribed, the pre-miRNAs will be further processed to give rise to the following mature miRNAs: VEGF miRNA: sense strand 5’-ACAGCAGAAAGTTCATGGTT-3’, antisense strand 5’-AACCATGATTTCTGCTGT-3’; scrambled miRNA: sense strand 5’-AAATGTACTGCGCGTGGAGA-3’, antisense strand 5’-TCTCCACGCAGTACATTT-3’. The silencing effects of these miRNAs were verified by qRT-PCR and Western blot analysis.

### Subcloning of Ang-2 into pcDNA3.1/(-)B and the transfection of cells

The Ang-2 gene was amplified by polymerase chain reaction (PCR) from the pBLAST49-hANGPT-2 plasmid (Invitrogen) and inserted into the pcDNA3.1(-)B vector (Invitrogen, San Diego, CA) at the BamH1 and EcoRI sites. Recombinant plasmids were identified by restriction enzyme analysis and DNA sequencing. The Ang-2 expression vector pcDNA3.1(-)B/Ang-2 was transfected into CNE2 cells, VEGF miRNA-transfected CNE2 cells, and scrambled miRNA-transfected CNE2 cells using a liposome transfection reagent. Parental CNE2 and CNE2 cells transfected with the empty pcDNA3.1(-)B vector were used as negative controls. Selective medium containing G418 (500 μg/mL) and blasticidin (2 μg/mL) was added 24 h later, and viable colonies were selected and expanded. Ang-2 and VEGF expression were evaluated by real-time RT-PCR and Western blot respectively. The expanded cells were harvested for *in vivo* animal experiments.

### Quantitative real-time RT-PCR

Total RNA was extracted from tumor cells or tissues using the TRIzol reagent (Invitrogen) according to the manufacturer's instructions. The RNA was further purified using the RNeasy Mini kit (Generay). cDNAs were synthesized using the Revert Aid First Strand cDNA synthesis Kit (Applied Fermentas). qRT-PCR was performed in a CFX connect Real-Time PCR System by using the SYBR Green PCR master mix (Applied Bio-Rad). The PCR cycle parameters were as follows: 50.0°C for 3 min, 95°C for 3 min, 40 cycles with denaturation at 95°C for 10 sec, annealing at 59.0°C for 20 sec, and extension at 72°C for 20 sec. All the PCR amplification was performed in triplicate and repeated in three independent experiments. The relative quantities of selected mRNAs in cell or tissue samples were normalized to that of GAPDH. The specific primer sequences used were as follows:

*HomoAng-2* (forward): 5′-ACTGGGAAGGGAATGAGGCTTACT-3′,

*HomoAng-2* (reverse): 5′-ATCAAACCACCAGCCTCCTGTT-3′,

*HomoVEGF* (forward): 5′-CTATCAGCGCAGCTACTGCCAT-3′,

*HomoVEGF* (reverse): 5′-GCACACAGGATGGCTTGAAGAT-3′,

*HomoGAPDH* (forward): 5′-AGAAGGCTGGGGCTCATTTG-3′, and

*HomoGAPDH* (reverse): 5′-AGGGGCCATCCACAGTCTTC-3′.

### Tumorigenicity assay

For the tumorigenicity assay, 5-week-old male nude mice were obtained from the Shanghai Slac Laboratory Animal Co. Ltd. and acclimated for 1 week. The mice were then caged in groups of four. The mice were randomly assigned to one of six treatment groups. The body weight at the time of assignment did not differ among the groups. After the viability of the cells had been verified by a trypan blue exclusion test, 2 × 10^6^ cells (in 0.2 mL Hank’s balanced salt solution) of each cell line were injected subcutaneously into the right flank of one nude mouse to expand the cell population *in vivo* (the first inoculation). Rapidly proliferating tumors were resected, and the necrotic tissue was removed. Viable tumor tissues of each treatment group from each mouse were cut into 2 mm^3^ pieces, and then subcutaneously inoculated into the right flanks of 9 new nude mice (the second inoculation). For the first inoculation, each mouse was injected with one of the following types of cells: parental CNE2 cells treated with only the liposome transfection reagent (group A: mock), empty-pcDNA3.1(-)B-transfected CNE2 cells (group B: control plasmid), Ang-2-transfected CNE2 cells (group C: Ang-2 plasmid), Ang-2-transfected and scrambled miRNA-transfected CNE2 cells (group D: Ang-2 + scrambled miRNA), Ang-2-transfected and VEGF miRNA-transfected CNE2 cells (group E: Ang-2 + VEGF miRNA), VEGF miRNA-transfected CNE2 cells (group F: VEGF miRNA). After the second inoculation, the animals were observed daily. The growth of each tumor was measured every three days. The tumor volume was calculated as V (cm^3^) = 1/2ab^2^ (a: length, b: width). All mice were sacrificed under sodium pentobarbital anesthesia on the 27^th^ day after the second inoculation. The tumors were harvested, weighed, and used for qRT-PCR, Western blot and immunohistochemical analyses.

### Detection of Ang-2 and VEGF protein expression

The Ang-2 and VEGF protein expression levels were determined by Western blot analysis. The lysates from tumor cells or tissue were subjected to SDS-PAGE, and the proteins were transferred to a nitrocellulose membrane (Millipore). The membrane was incubated with primary antibodies against Ang-2 (GTX100927, Gene Tex) or VEGF (9003-1-AP, Protein Tech), followed by incubation with a horseradish peroxidase-conjugated goat anti-rabbit IgG (BS13278, Bioworld). The membrane was incubated with the enhanced chemiluminescence plus detection reagent and exposed to film. The densitometric intensities of the bands were then compared.

### Histological and immunocytochemical examination

Solid tumors from mice were embedded in paraffin wax after dehydration using an ethanol series. A rabbit polyclonal antibody against α-smooth muscle actin (α-SMA) was purchased commercially (Proteintech Group Inc). Formaldehyde-fixed, paraffin-embedded tissue sections were deparaffinized in xylene and rehydrated in alcohol. Then, the sections were incubated in 3% hydrogen peroxide to block endogenous peroxidase activity. Each slide was incubated with normal goat serum for 30 min at room temperature and then with the α-SMA antibody overnight at 4°C. After incubation with a biotinylated mouse anti-goat IgG (working dilution, 1:200) for 30 min at room temperature, each slide was rinsed with PBS and incubated with an avidin-biotin peroxidase complex for 30 min at 37°C. The peroxidase was visualized using a 3,3’-diaminobenzidine tetrahydrochloride solution, and the sections were counterstained with methyl green. α-SMA is a marker of smooth muscle cells, which normally associate with endothelial cells to form mature vascular structures. In this study, the immunohistochemical staining of α-SMA was used to assess the maturity of the vessels.

### Tumor vascularity

Solid tumors from different groups of injected mice were embedded in paraffin wax after dehydration. Tumor pieces were each cut into 5 μm consecutive paraffin sections and stained with the EC-specific marker CD34 (Proteintech Group Inc., 1:200), followed by detection using the avidin-biotin complex method and 3,3’-diaminobenzidine according to the instructions. The microvessel density (MVD) was calculated according to the method reported by Weidner et al. [20]. Briefly, low-power light microscopy (magnification × 100) was used to scan the often heterogeneous tumor sections to identify the areas with the highest levels of neovascularization. Any individual endothelial cell or endothelial cell cluster that was positive for CD34 and was clearly separate from all nearby clusters was considered a countable microvessel. Individual microvessels were counted in the three areas with the highest vascular density in a × 200 field. The count was expressed as the mean number of vessels in these areas and was recorded as the MVD. The MVD was determined by two separate investigators.

### Statistical analysis

The values were expressed as the means ± SD. Normality of the data was tested using SPSS, revealing all the experimental data as normally distributed data. The significance of the differences between experimental groups was determined by ANOVA in SPSS version 11.0. P values of <0.05 were considered statistically significant.

## Results and discussion

### Parental CNE2 cells express a relatively high level of VEGF, which was silenced by VEGF-specific miRNA

**Figure 1:**
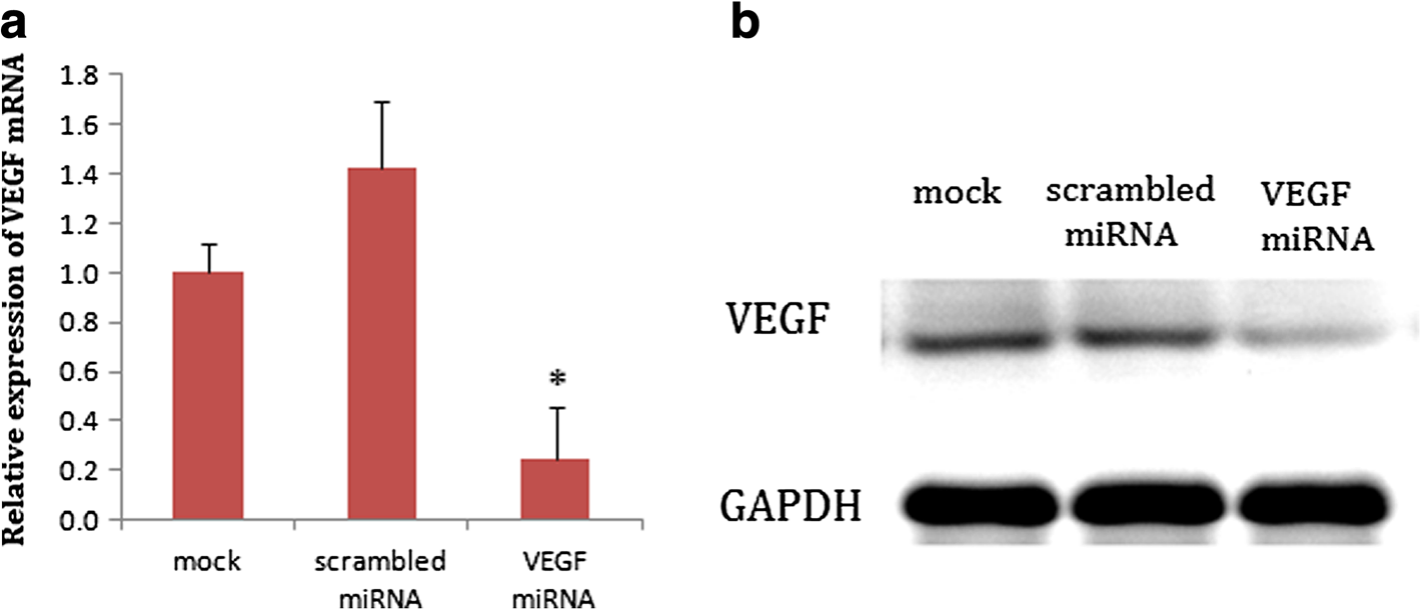
**Effects of VEGF silencing on CNE2 cells.** CNE2 cells were treated with the transfection reagent only (mock), a scrambled miRNA (scrambled miRNA), or a VEGF-specific miRNA (VEGF miRNA). **(a)**: Real-time RT-PCR demonstrated low VEGF mRNA expression in VEGF-interfered CNE2 cells. *P < 0.05, compared with the mock and scrambled miRNA groups. **(b)**: Western blotting demonstrated lower VEGF protein expression in the VEGF-interfered CNE2 cells.

VEGF-specific miRNA was transfected into CNE2 cells using Lipofectamine 2000 and cells stably integrating the pre-miRNA oligo sequences were selected for the following experiments. We compared the expression of VEGF between the VEGF miRNA-transfected CNE2 cells and the control cells using qRT-PCR and Western blot analyses. The results showed the expression of VEGF was higher in the parental CNE2 cells and scrambled miRNA-transfected CNE2 cells but significantly lower in the VEGF miRNA-transfected CNE2 cells (*p* < 0.05, Figure 1), with a 76% reduction of endogenous VEGF, indicating that the VEGF miRNA effectively interfered with VEGF expression.

### Successful expression of ectopic Ang-2 in CNE2 and VEGF-interfered CNE2 cells

**Figure 2:**
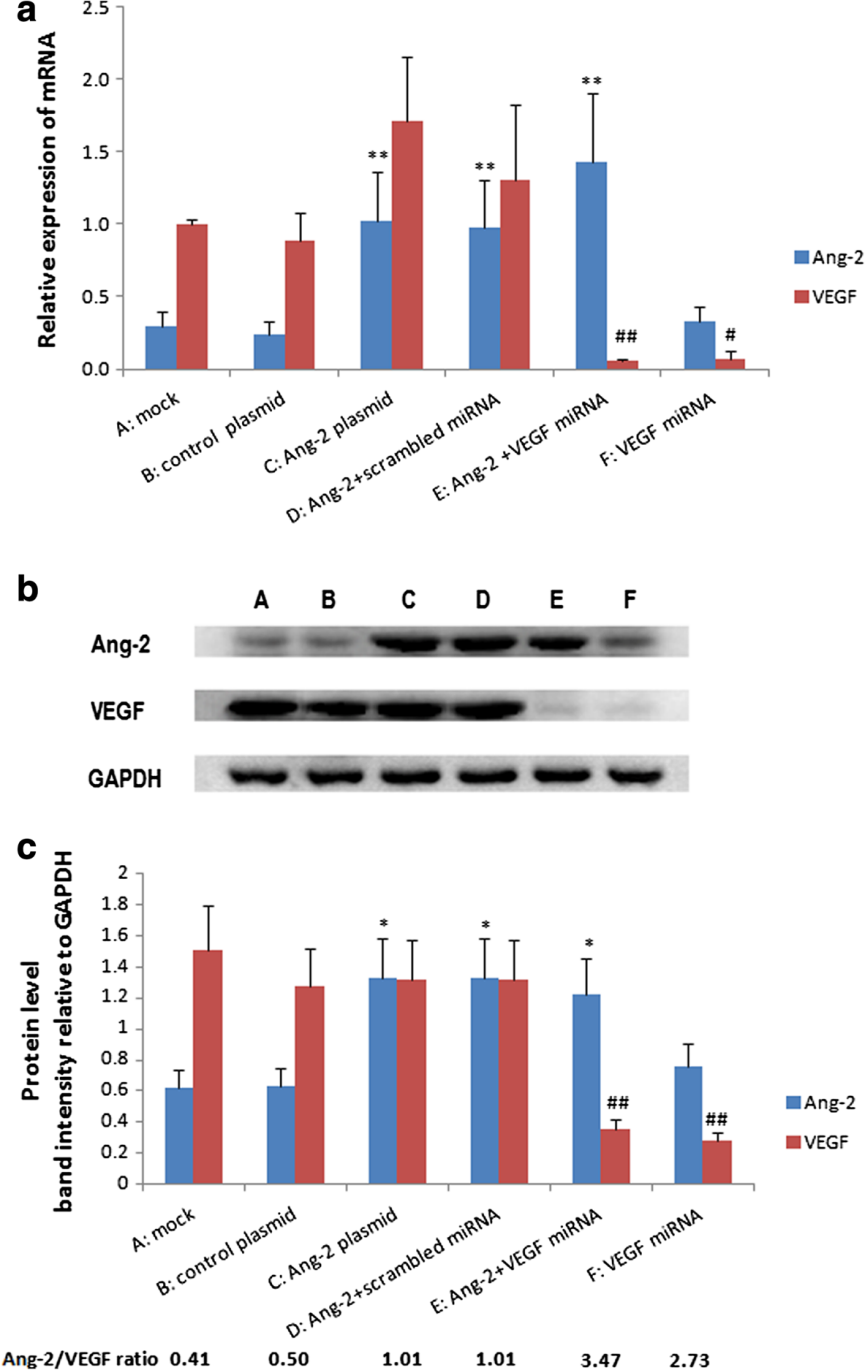
**Expression levels of Ang-2 and VEGF in different engineered CNE2 cell groups. (a)**: mRNA expression was determined by qRT-PCR. All the PCR amplification was performed in triplicate and repeated in three independent experiments. The relative quantities of selected mRNAs in cell samples were normalized to that of GAPDH. Higher Ang-2 mRNA expression was observed in C, D, and E (**P < 0.01, *vs.* B, no significant difference among A, B and F). Lower VEGF mRNA expression was observed in group E and F (## P < 0.01, # P < 0.05, *vs.* A, B, C, D). **(b)**: Western blots of the above samples. **(c)**: Densitometric quantitation of the band intensities shown in **(b)**. The densitometric intensity of the target band in each lane relative to the intensity of the GAPDH band from the same sample was calculated, and plotted. Data were expressed as means ± SD of three independent experiments. The level of total Ang-2 protein in the Ang-2-transfected group C,D,E was significantly higher than that in control group B (*P < 0.05 *vs.* B; no significant difference among A, B and F), and the level of endogenous VEGF protein in the VEGF miRNA-transfected group E,F was significantly decreased (## *P <* 0.01, *vs.* A, B, C, D). The ratio of Ang-2/VEGF was significantly higher in E and F than in other groups.

The purified full-length cDNA for Ang-2 was subcloned into the pcDNA3.1(-)B vector using T4 DNA ligase. The insert was verified by DNA sequencing as human Ang-2 (accession number: NM001147).The pcDNA3.1(-)B/Ang-2 vector was then transfected into CNE2 and VEGF-silenced CNE2 cells using liposomes. Stable clones were selected. A higher level of Ang-2 in Ang-2-transfected cells was confirmed by real-time quantitative RT-PCR and Western blot. The levels of Ang-2 and VEGF expression and the ratio of Ang-2/VEGF in the six groups of cells were shown in Figure 2.

### Overexpression of Ang-2 had no effect on CNE2 tumor growth but greatly suppressed growth when the VEGF level was low

**Figure 3:**
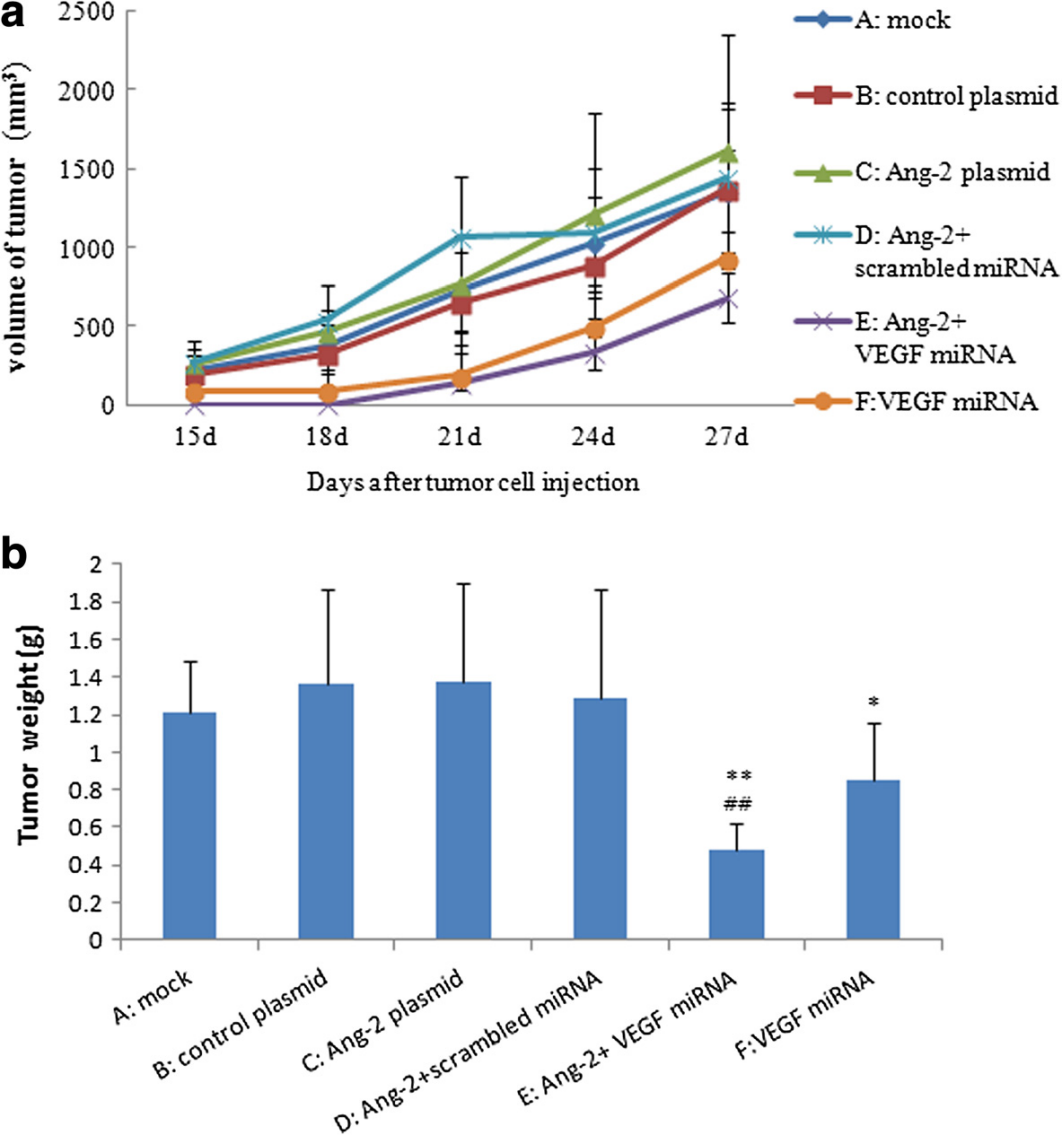
**Effect of Ang-2 overexpression on CNE2 tumor growth under different VEGF level.** Six groups of engineered CNE2 cells with different Ang-2 and VEGF levels were subcutaneously inoculated into six groups of nude mice (A-F) respectively. Tumor volumes were calculated dynamically **(a)**, and tumors were weighed **(b)** when the mice were sacrificed on the 27^th^ day after inoculation. There was no significant difference on tumor development among A,B,C. (*P* > 0.05), while *E* and *F* exhibited greatly suppressed tumor growth compared with A,B,C,D (**P < 0.01, *P < 0.05 vs *A,B,C,D, ## P <* 0.01 vs *F*). *E* had higher inhibitory effects than *F* on tumor growth.

Small tumor pieces derived from the first round of inoculation with one of the six groups of CNE2 cells - mock, control plasmid, Ang-2 plasmid, Ang-2 + scrambled miRNA, Ang-2 + VEGF miRNA and VEGF miRNA - were inoculated subcutaneously into the flanks of nude mice to establish CNE2 xenografts. After the second inoculation, tumor growth was monitored daily and compared among the different groups. No obvious side effects were observed during the experimental period. The group transgenically expressing Ang-2 had no obvious effect on CNE2 tumor growth compared with the mock and control plasmid transfected groups. However, the transgenic expression of Ang-2 greatly suppressed tumor growth in the VEGF-silenced CNE2 cells. VEGF miRNA group also had a prominent inhibitory effect on tumor growth, but Ang-2 + VEGF miRNA group which had a higher Ang-2/VEGF ratio exhibited an even higher degree of tumor suppression than the VEGF miRNA group (Figure 3a).

All mice were sacrificed on the 27^th^ day after the second inoculation. qRT-PCR and Western blot analysis of the harvested tumors revealed higher Ang-2 expression in the Ang-2-transfected groups and lower VEGF expression in the VEGF-silenced CNE2 group (*P <* 0.05, Additional file 1: Figure S1). The weights of the transplanted tumors on treatment day 27 from the six groups were presented in Figure 3b. There were no significant differences between the Ang-2 transgenic group and the controls (*P* > 0.05). However, Ang-2 expression combined with VEGF miRNA resulted in a greatly decreased tumor weight compared with those of the other five groups including VEGF miRNA group (*P <* 0.05, Figure 3b).

### Overexpression of Ang-2 decreased tumor angiogenesis when the VEGF level was low

**Figure 4:**
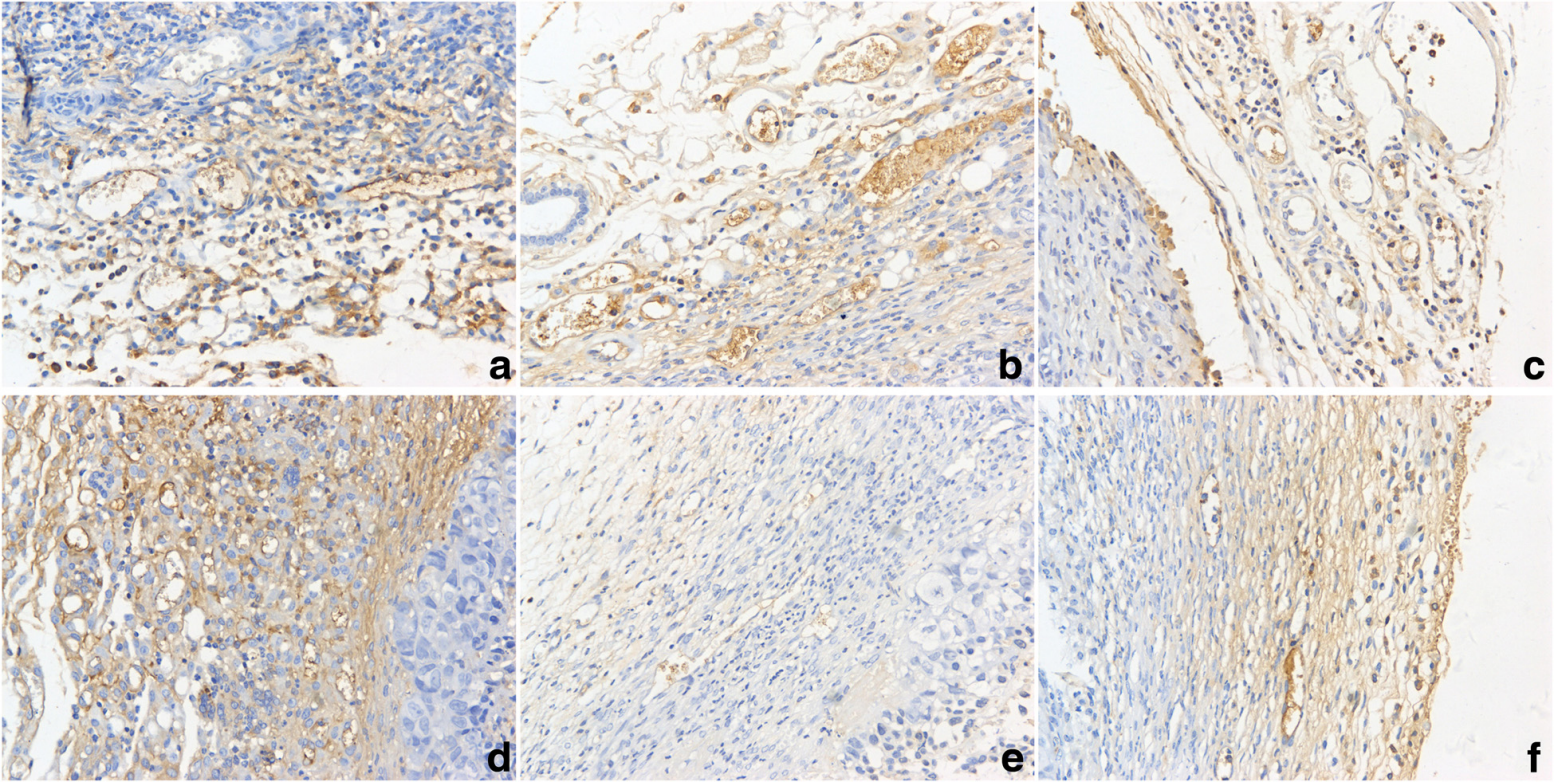
**Images for CD34 staining in harvested tumor tissues with microvessel density measured.** It showed that there were higher microvessel density (MVD) in A,B,C,D group, while lower MVD in E and F (VEGF silenced group). **a**: mock, **b**: control plasmid, **c**: Ang-2 plasmid, **d**: Ang-2 + scrambled miRNA, **e**: Ang-2 + VEGF miRNA, **f**: VEGF miRNA. Original magnifications: 200× for **a**,**b**,**c**,**d**,**e**,**f**.

To determine the number of microvessels, CD34 immunoreactivity was detected in vascular endothelial cells. Microvessels appeared as brown capillaries or small clusters standing out from other tissue. The MVDs in tumors derived from the different types of cells were analyzed. There was no significant difference between the Ang-2-transfected CNE2 tumors and the control tumors (*P* > 0.05). However, the tumors derived from the VEGF-silenced CNE2 groups exhibited a significantly decreased MVD relative to the other groups (Table 1, Figure 4).

**Table 1:** Immunohistochemical staining for CD34 was used to determine the MVD in harvested tumors derived from the different CNE2 groups (MVD in 200× field)

**Group**	**MVD**
A: mock	22.89 ± 3.15
B: control plasmid	23.04 ± 3.61
C: Ang-2 plasmid	21.67 ± 3.51
D: Ang-2 + scrambled miRNA	21.11 ± 2.97
E: Ang-2 + VEGF miRNA	9.48 ± 2.23** ##
F: VEGF miRNA	12.04 ± 3.03**

**: P < 0.01 vs. A,B,C,D; ## P < 0.01 *vs F.*

no significant difference among A,B,C (*P* > 0.05), by ANOVA.

**Figure 5:**
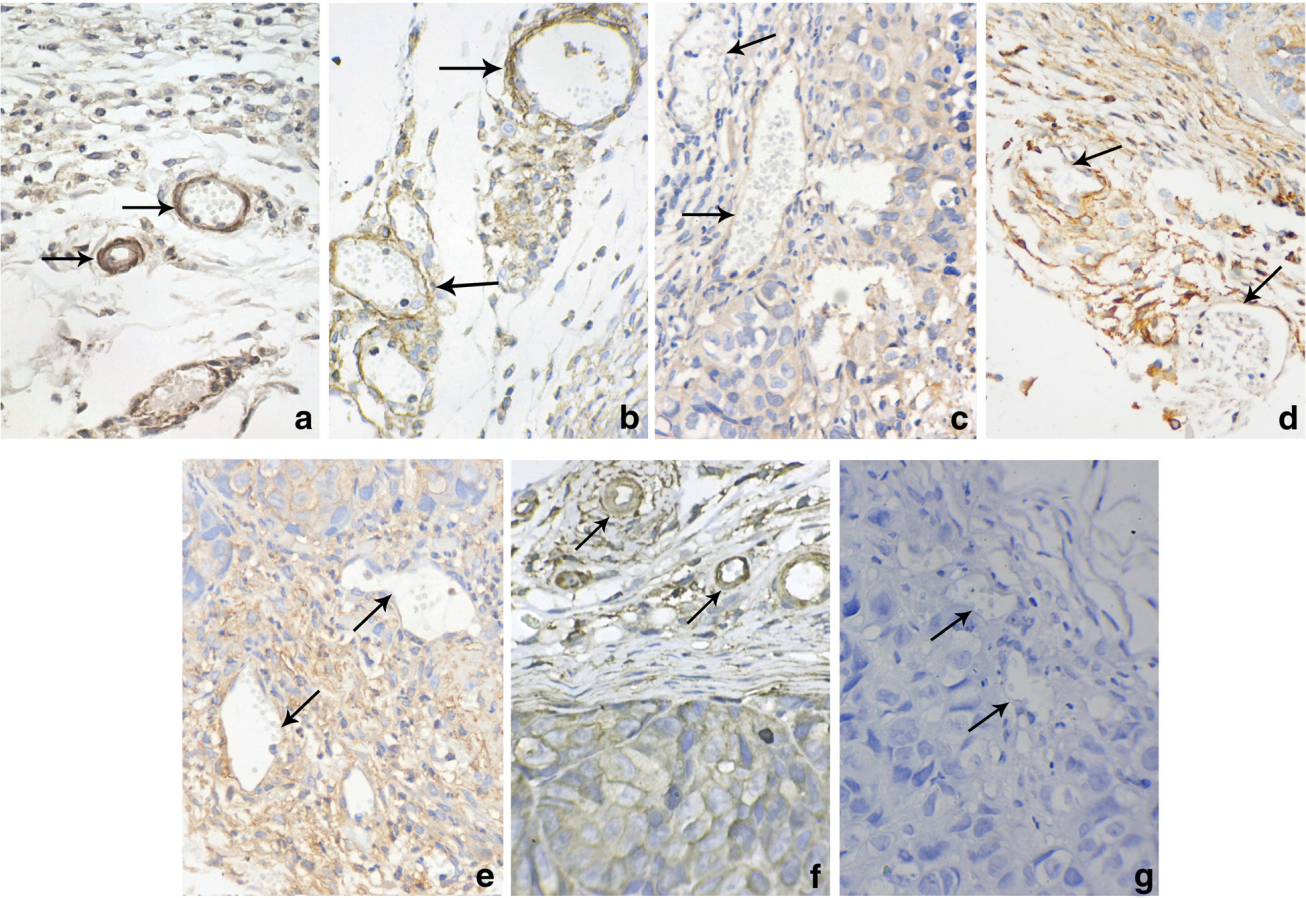
**Immunohistochemical staining for α-SMA in harvested tumor tissues.** The results showed that there were fewer α-SMA-positive cells around endothelial cells in the Ang-2-transfected groups **(c,d,e)** than in non- Ang-2-transfected groups **(a,b,f)**. In addition, this staining showed vessels in **c,d,e** displayed an aberrant structure characterized by distorted and dilated lumina (vessel indicated by arrow). **a**: mock, **b**: control plasmid, **c**: Ang-2 plasmid, **d**: Ang-2 + scrambled miRNA, **e**: Ang-2 + VEGF miRNA, **f**: VEGF miRNA, **g**: negative control. Original magnifications: 400× for **a**,**b**,**c**,**d**,**e**,**f**,**g**.

To evaluate the effects of Ang-2 overexpression on the maturation of tumor vessels, α**-**SMA was detected by immunohistochemical staining. There were typically fewer α**-**SMA-positive cells around the tumor vessels in the Ang-2-transfected groups than in the mock and control plasmid groups. Tumor vessels in Ang-2-transfected groups displayed aberrant structures characterized by distorted, dilated or narrow vessel lumina (Figure 5). This result indicated that the vessels in the Ang-2-transfected tumors were immature.

Ang-2 plays an important role in angiogenesis during the development and growth of human cancers. This protein increases the permeability of the vascular endothelial layer of mature blood vessels and makes the vessel more plastic. Ang-2 modulates tumor angiogenesis in a cooperative manner with other angiogenic factors, especially VEGF. Although almost all tumors exhibit upregulated Ang-2 expression, the roles of Ang-2 in tumor angiogenesis were found to be complex and variable, and its exact effects depend on the tumor model investigated.

Past studies have shown that overexpression of Ang-2 augments tumor angiogenesis and growth and is associated with a poor prognosis. For example, Ang-2-expressing MKN-7 gastric carcinoma cells developed tumors with hypervascularity and a high metastatic potential [21]. When Tanaka et al. [22] injected Ang-2 overexpressing human HuH7 hepatocellular cancer cells into the livers of nude mice, all of the mice exhibited extensive bleeding at death, after the formation of large tumors. The same result was found for neuroendocrine tumors [4] and colon cancer [6]. These findings support the hypothesis that overexpression of Ang-2 can lead to increased tumor growth and hypervascularity.

In contrast, there were also data suggesting that the specific induction of Ang-2 in gliomas, mammary carcinomas, and lung carcinomas inhibits tumor growth and metastasis [7-9]. By impairing the pericyte coverage of the tumor vasculature, Ang-2 induced massive regression of the tumor vascular and exacerbated tumor hypoxia, effects that significantly inhibited tumor angiogenesis, promoted tumor apoptosis, and suppressed tumor growth [10].

Interestingly, some studies indicated that Ang-2 had no significant effect on tumor growth. For example, Sung-Suk Chae et al. [23] showed that ectopic expression of Ang-2 had no effect on U87 glioma tumor growth. Study from H Yoshiji et al. [24] also indicated that overexpression of Ang-2 did not increase hepatocellular carcinoma development. Our current results are in line with their findings.

Why does Ang-2 have different effects on different tumor types or models? Our results revealed that the level of Ang-2 relative to VEGF (i.e., the ratio of Ang-2/VEGF) rather than its absolute level might be critical in tumor suppression. It is well established that tumor angiogenesis is regulated by a variety of proangiogenic factors, among which, VEGF and Ang-2 are the two most important factors that seem to play coordinated roles in vascular development to support tumor growth. The Ang-2-Tie2 interaction has been shown to increase the permeability of the vascular endothelial layer of mature blood vessels by disrupting interactions between endothelial cells (ECs) and peri-ECs, inducing a localized region of vessel plasticity. This plasticity facilitates EC migration, exposing ECs to proliferation signals from angiogenesis inducers, especially VEGF [25]. Initially, when there is a low level of endogenous VEGF expression, Ang-2 destabilises vessels by removing pericytes and causing irreversible loss of vascular structures and relative hypoxia, which may collectively suppress tumor growth. However, hypoxia can drive the release of VEGF, and in the presence of VEGF, Ang-2 may induce an angiogenic response and promote vascular sprouting from disrupted vessels, and eventually, high expression of Ang-2 and VEGF leads to robust angiogenesis. Therefore, it is reasonable to speculate that the relative level of Ang-2 versus VEGF is important in controlling the angiogenic switch and tumor growth, and up-regulating Ang-2 while down- regulating VEGF simultaneously may be an effective therapeutic approach for suppressing tumor growth. In different tumor models or the same tumor at different stages, the ratio of Ang-2/VEGF may vary, which could offer an explanation for the different effects of Ang-2 and its stage-dependent roles in tumor growth. In our current study, when VEGF expression was effectively silenced by miRNA, overexpression of Ang-2 accelerated vessel regression and suppressed tumor growth. There were fewer α-SMA-positive cells around the vessels in Ang-2-transfected tumors, and vessels displayed aberrant structures characterized by distorted ,dilated or narrow vessel lumina, but there were no significant difference in microvessel density (MVD) between Ang-2-transfected group and the control. However, Ang-2 overexpression together with VEGF-silencing significantly decreased MVD indicating that the immature vessels induced by Ang-2 were partially regressed in the low level of VEGF.

## Conclusions

In summary, our study showed that the effects of Ang-2 overexpression on nasopharyngeal CNE2 tumor growth are dependent on the VEGF expression level. Specifically, Ang-2 can greatly inhibit tumor growth in the low level or absence of VEGF, and the Ang-2/VEGF ratio is negatively correlated with tumor growth. These results may have the following important implications: firstly, It is now widely recognized that VEGF and Angs are two central endothelium specific growth families coordinating tumor angiogenesis, but how they operated are still unclear. Our data indicated combination of Ang-2 and VEGF may provide a more reliable prognostic index for some cancers. Secondly, for the first time, our data revealed the importance of a high ratio of Ang-2/VEGF in suppressing tumor growth, and clearly demonstrated the advantageous effects of simultaneously elevating Ang-2 level and lowering VEGF level, and potentially opened new avenues for angiogenic factor-based anticancer therapy. However, our conclusions were largely based on experiments employing only the CNE2 cell line, further studies are required to determine whether the combined targeting of Ang-2 and other angiogenic cytokines in a variety of primary cancer tissues and cancer cell lines can be used as a safe and effective therapeutic intervention for the treatment of cancer.

## Abbreviations

Ang: Angiopoietin; Ang-2: Angiopoietin-2; VEGF: Vascular endothelial growth factor; ECs: Endothelial cells; α-SMA: α-smooth muscle actin; NPC: nasopharyngeal carcinoma; qRT-PCR: Reverse transcription-polymerase chain reaction; MVD: Microvessel density; GAPDH: Glyceraldehyde-3-phosphate dehydrogenase.

## Competing interests

We have no financial and non-financial competing interests.

## Authors’ contributions

H-HC conceived and designed of the study, carried out the gene transfection, participated in the sequence alignment, reviewed literatures and wrote the article. B-QW carried out animal experiment. K-JC carried out real-time RT-PCR and Western blot, participated in gene transfection, and performed the statistical analysis. H-YL carried out the immunoassays. S-QW participated in its design and coordination, helped to draft and finally revised the manuscript. Y-YL participated in the sequence alignment. We all read and approved the final manuscript.

## Authors’ information

Hai-Hong Chen: Doctor of Medicine(M.D.). graduated in 2007, worked in the 1^ST^ Affiliated Hospital of Zhejiang University as a Otolaryngological doctor, Specialized in tumor angiogenesis research.

Ke-Jia Cheng: Master degree, graduated in 2006, now is a doctoral candidate, specialized in head and neck oncology research.

Shen-Qing Wang: Professor, worked in the 1^st^ Affiliated Hospital of Zhejiang University as a Otolaryngological doctor, majored in head and neck oncology surgery.

## Supplementary Material

Additional file 1: Figure S1 Expression levels of Ang-2 and VEGF in tumor tissues resected from mice inoculated with different engineered CNE2 cells. (*a):* mRNA expression was determined by qRT-PCR. Higher Ang-2 mRNA expression was observed in C, D, and E (*P< 0.05 *vs.* B, no significant difference among A,B and F). Lower VEGF mRNA expression was observed in group E and F (# P < 0.05, *vs.* A, B, C, D). *(b)*: Western blots of the above samples. *(c)*: Densitometric quantitation of the band intensities shown in *(b)*. The level of total Ang-2 protein in the Ang-2-transfected group C,D,E was significantly higher than that in control group B (*P<0.05, *vs.* B; no significant difference among A, B and F), and the level of endogenous VEGF protein in the VEGF miRNA-transfected group E,F was significantly decreased (## *P<*0.01, *vs.* A, B, C, D). The ratio of Ang-2/VEGF was significantly higher in E and F than in other groups. Click here for file
