# Supplementary figures and images for: Effect of the vascular endothelial growth factor expression level on angiopoietin-2-mediated nasopharyngeal carcinoma growth
Source: Vasc Cell. 2014 Mar 1;6:4. doi: 10.1186/2045-824X-6-4 (PMC4015607; doi:10.1186/2045-824X-6-4)

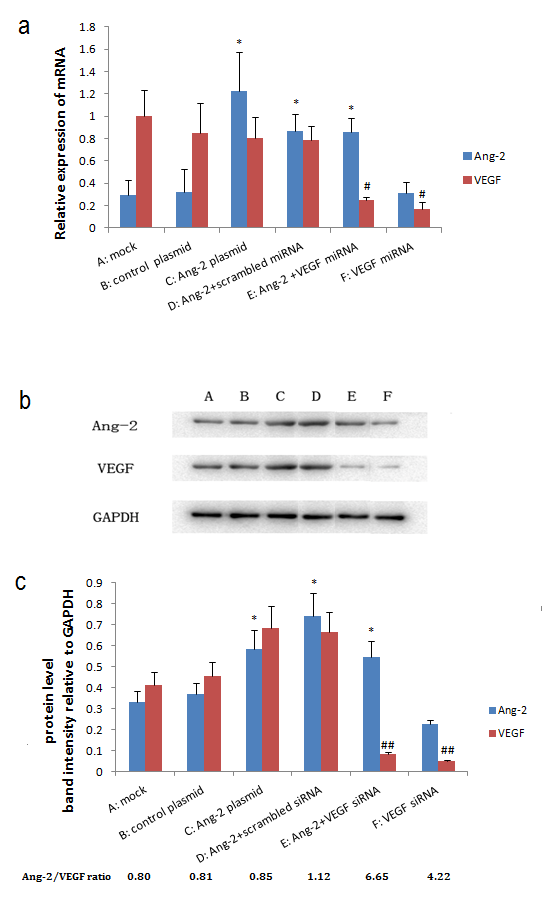

Supplement: Additional file 1: Figure S1 — Expression levels of Ang-2 and VEGF in tumor tissues resected from mice inoculated with different engineered CNE2 cells. (a): mRNA expression was determined by qRT-PCR. Higher Ang-2 mRNA expression was observed in C, D, and E (*P< 0.05 vs. B, no significant difference among A,B and F). Lower VEGF mRNA expression was observed in group E and F (# P < 0.05, vs. A, B, C, D). (b): Western blots of the above samples. (c): Densitometric quantitation of the band intensities shown in (b). The level of total Ang-2 protein in the Ang-2-transfected group C,D,E was significantly higher than that in control group B (*P<0.05, vs. B; no significant difference among A, B and F), and the level of endogenous VEGF protein in the VEGF miRNA-transfected group E,F was significantly decreased (## P<0.01, vs. A, B, C, D). The ratio of Ang-2/VEGF was significantly higher in E and F than in other groups. [file 2045-824X-6-4-S1.tiff]
